# Editorial: The relationship between nutrition and frailty/multimorbidity: prevention and clinical nutritional management

**DOI:** 10.3389/fnut.2026.1821896

**Published:** 2026-03-27

**Authors:** Akio Shimizu, Fumiya Kawase, Tatsuro Inoue

**Affiliations:** 1 Department of Rehabilitation Medicine, Mie University Graduate School of Medicine, Tsu, Japan; 2 Department of Sports and Health Sciences, Graduate School of Biomedical Engineering, Tohoku University, Sendai, Japan; 3 Department of Physical Therapy, Niigata University of Health and Welfare, Niigata, Japan

**Keywords:** body composition, dietary patterns, frailty, malnutrition, multimorbidity, nutritional assessment, nutritional management, older adults

The global population of older adults is expanding at an unprecedented pace, with projections indicating that by 2050, one in six people worldwide will be aged 65 years or older. This demographic shift has drawn significant attention to the interconnected challenges of aging, including the high prevalence of frailty, multimorbidity, and malnutrition in older adults. Frailty—a multidimensional syndrome characterized by diminished physiological reserves and heightened vulnerability to stressors (1)—and multimorbidity, the co-occurrence of two or more chronic conditions, represent major public health concerns affecting healthcare costs, functional independence, and mortality risk in aging populations (2, 3). The relationship between malnutrition and these age-related syndromes is increasingly recognized, yet often overlooked in clinical practice. Malnutrition can accelerate functional decline, compound the metabolic consequences of chronic diseases, and significantly worsen health outcomes, even in seemingly stable populations (4). Understanding this triad of nutritional status, frailty, and multimorbidity is essential for developing effective prevention and management strategies.

This Research Topic comprises 19 original contributions (excluding one correction notice), encompassing original research articles, systematic reviews and meta-analyses, and a case report, published across Frontiers in Nutrition, Frontiers in Medicine, and Frontiers in Public Health. These diverse contributions span multiple dimensions of the nutrition–frailty–multimorbidity relationship, from epidemiological investigations of disease prevalence to mechanistic studies exploring biomarkers and nutritional indices, and from dietary pattern analyses to targeted clinical interventions. Taken together, they provide a comprehensive evidence base that extends across prevention, assessment, and management of these interconnected conditions.

Malnutrition screening and detection in vulnerable populations form a critical foundation for improving outcomes. Zheng, Zhang et al. investigated malnutrition prevalence and clinical consequences in older adults hospitalized with community-acquired pneumonia, demonstrating that malnutrition affected 17.6% of patients and independently predicted prolonged hospital stay, higher in-hospital mortality, and increased readmission risk. This observation underscores the importance of prompt nutritional assessment during acute illness. Coelho-Júnior et al. applied the Global Leadership Initiative on Malnutrition (GLIM) criteria to characterize malnutrition among community-dwelling Italian octogenarians and found a significant association between GLIM-defined malnutrition and fall history. Meanwhile, Zhu et al. conducted a systematic review and meta-analysis demonstrating that cognitive frailty affects approximately 26% of Chinese older adults and is strongly associated with malnutrition (OR = 4.23), indicating that this relationship may be a critical intervention target. Scisło et al. demonstrated that polypharmacy among long-term care residents is negatively correlated with nutritional status and importantly, showed that targeted oral nutritional supplementation can improve measurable nutritional parameters, highlighting the potential for nutritional interventions even in polymedicated populations.

Beyond clinical assessment alone, validated nutritional indices and biomarkers have emerged as important predictors of frailty and adverse outcomes. Zhou et al. analyzed the prognostic nutritional index (PNI) using large NHANES cohorts, revealing non-linear relationships between PNI and all-cause cardiovascular mortality with important threshold effects. Xu et al. demonstrated that the body roundness index is positively associated with frailty prevalence in older Americans, suggesting that body shape and composition patterns have independent predictive value beyond traditional anthropometric measures. Zheng, Zhou et al. characterized a non-linear protective relationship between the geriatric nutritional risk index (GNRI) and overactive bladder in elderly individuals, with inflammatory markers serving as a crucial mediating mechanism. Pan et al. reported a U-shaped relationship between non-HDL-cholesterol and frailty status, which they validated across multiple large databases (NHANES and CHARLS) using machine learning algorithms to improve robustness. Li et al. identified a novel non-linear correlation between the hemoglobin-to-red blood cell distribution width ratio and albuminuria, suggesting that hematologic composition may inform understanding of kidney function and nutritional status.

Dietary patterns and gut microbiota have also emerged as important determinants of frailty risk. Using the Dietary Index for Gut Microbiota (DI-GM) derived from NHANES data, Yang et al. found that inflammatory parameters significantly mediate the inverse association between higher DI-GM scores and reduced frailty risk, suggesting that a gut microbiota-favorable diet may protect against frailty through anti-inflammatory pathways. Similarly, Lei et al. identified body mass index as an additional mediating factor in the DI-GM–frailty relationship, highlighting the complex interplay among dietary quality, body composition, and frailty development. Ouyang et al. assessed dietary quality in Chinese patients with chronic kidney disease and found that adherence to dietary recommendations is low overall and worsens as CKD stage severity increases, suggesting that patients faced both multimorbidity and nutritional challenges require specific dietary guidance and support.

The investigation of micronutrient status, body composition, and metabolic markers continues to reveal important mechanistic insights. Chan et al. examined the relationships among serum vitamin D concentration, the systemic immune-inflammation index, and lifestyle factors, providing mechanistic evidence that vitamin D deficiency is associated with elevated systemic chronic inflammation markers in Chinese populations. Vicinanza et al. demonstrated that insulin-like growth factor I (IGF-I) mediates the effect of vitamin D on fat-free mass in geriatric outpatients, suggesting potential mechanisms for vitamin D's role in preventing frailty progression. Liu et al. identified a two-phase linear relationship between vitamin D and vitamin A, with an important threshold effect, suggesting that optimal micronutrient balance may be essential during childhood and growth. Omaña-Guzmán et al. demonstrated in a prospective cohort study that both undernutrition risk and obesity increase the likelihood of developing osteosarcopenia in older Mexican adults, indicating that poor nutritional status affects not only muscle but also bone quality and strength. Wen et al. showed that malnutrition partially explains the relationship between reduced handgrip strength and asthma risk, suggesting that sarcopenia and nutritional deficiency may contribute to broader systemic vulnerabilities in respiratory and metabolic health.

This Research Topic also includes contributions addressing clinical nutritional interventions. Ren et al. conducted a comprehensive systematic review and meta-analysis of beta-hydroxy-beta-methylbutyrate (HMB) supplementation in critically ill patients, finding that despite mechanistic promise, HMB did not significantly improve mortality or clinical outcomes—a valuable negative finding that advances evidence-based practice by clarifying which interventions require further investigation and which may not translate to clinical benefit. Chen et al. presented a detailed case report demonstrating innovative enteral nutrition delivery using electromagnetic navigation-guided nasojejunal tube placement in an elderly critically ill patient with complex anatomy and gastroesophageal reflux disease, illustrating how creative clinical approaches can overcome challenges in nutritional support.

In summary, the contributions to this Research Topic collectively illuminate the multifaceted relationship between nutrition, frailty, and multimorbidity. A notable strength of this Research Topic is its geographical and methodological diversity, encompassing cohort studies, cross-sectional surveys, meta-analyses, and case reports from China, the United States, Europe, Mexico, and East Asia. The frequent use of large, well-established population-based databases such as NHANES, CHARLS, and SHARE further strengthens the epidemiological evidence base. Several cross-cutting themes emerge: the consistent presence of non-linear relationships between nutritional indicators and health outcomes, the central role of inflammation as a mediating pathway, and the importance of validated screening and assessment tools. However, several important limitations should be noted. The majority of studies in this Research Topic used cross-sectional designs, which preclude causal inference regarding the directionality of the nutrition–frailty relationship. Furthermore, longitudinal prospective studies and randomized controlled trials of nutritional interventions remain underrepresented, particularly those targeting multimorbid populations. Future research must prioritize bidirectional analysis of nutrition and frailty trajectories, well-designed trials of targeted interventions in multimorbid populations, and the integration of emerging approaches such as gut microbiota-informed dietary strategies. We hope this Research Topic serves researchers, clinicians, and public health professionals seeking to advance nutritional care and improve outcomes for older adults at risk of frailty and multimorbidity.
